# Are lichens biocompasses? Revisiting an old prediction using new techniques

**DOI:** 10.1111/plb.70055

**Published:** 2025-05-19

**Authors:** B. B. S. Nunes, A. Braga, M. E. M. Marques, I. Lopes, J. C. F. Cardoso

**Affiliations:** ^1^ Programa de Pós‐Graduação em Ecologia, Conservação e Biodiversidade Universidade Federal de Uberlândia Uberlândia Minas Gerais Brazil; ^2^ Instituto de Geografia, Geociências e Saúde Coletiva Universidade Federal de Uberlândia Uberlândia Minas Gerais Brazil; ^3^ Centro do Biociências e Biotecnologia Universidade Estadual do Norte Fluminense Darcy Ribeiro Rio de Janeiro Brazil; ^4^ Instituto de Biociências Universidade Federal do Rio Grande do Sul Porto Alegre Rio Grande do Sul Brazil; ^5^ Departamento de Biologia Geral Universidade Federal de Viçosa Viçosa Minas Gerais Brazil; ^6^ Programa de Pós‐Graduação em Ecologia, Conservação e Manejo da Vida Silvestre Universidade Federal de Minas Gerais Belo Horizonte Minas Gerais Brazil

**Keywords:** Circular statistics, environmental filters, epiphytic plants, radial orientation

## Abstract

- Abiotic factors influence not only vertical distributions of epiphytes, but also their radial positioning, such as around tree trunks. Due to planetary patterns of solar incidence, the equator‐facing trunk portions receive direct sunlight, creating restrictive microclimatic conditions. Conversely, pole‐facing portions remain shaded, providing optimal conditions for some epiphytes. Since trunk microhabitats are continuous, the distribution of epiphytes involves directional (compass‐derived) data, requiring circular analytical methods. However, such assessments remain unexplored for avascular epiphytes.
- In the southern hemisphere, we investigated radial distribution of all lichen crustose and foliose thalli occurring on concentric trunks of a *Syagrus oleracea* population. We hypothesized that: (1) both lichen types will orient southward, the optimal zone; (2) lichen types do not differ in preferred direction, indicating a stronger microenvironmental than interaction filter; and (3) other variables (height, phorophyte size, canopy cover) do not affect orientation, which is primarily governed by azimuth. We address these questions using circular statistics, including preferred direction tests, between‐sample comparisons, and linear‐circular regressions.
- We surveyed 845 lichen thalli (180 crustose, 665 foliose). The lichens were non‐uniformly distributed and oriented southward, with similar distributions. Radial orientation was unaffected by height, canopy cover, or phorophyte size, confirming azimuth as the primary determinant of orientation.
- These results demonstrate how abiotic conditions, driven by planetary patterns, indirectly determine lichen niche occupation on a fine radial dimension of trunks, offering new insights into epiphyte ecology.

## Graphical abstract

The equator‐facing portions of tree trunks receive direct sunlight and create restrictive microclimate conditions for lichens that orient preferentially towards the Earth's poles where trunks are shaded, indicating an optimal zone at the fine‐scale radial dimension.

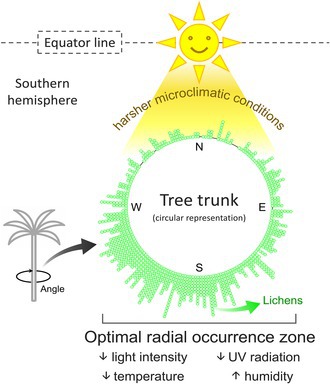

## INTRODUCTION

Because they are sessile, photosynthesizing organisms must cope with abiotic stressors, such as light intensity, temperature, and wind (Ashraf & Harris 2013; Hurtado *et al*. 2019). After overcoming dispersal and environmental filters, autotrophs still have an interaction filter, including biotic factors, such as ecological interactions (Kraft *et al*. 2015; Lõhmus *et al*. 2023). Both abiotic and biotic factors impact establishment, photosynthetic efficiency, growth, development, and survival of autotrophs, ultimately determining their distribution (Ashraf & Harris 2013; Hurtado *et al*. 2019; Llewellyn *et al*. 2020). This distribution can be restricted at macro‐ and micro‐scales (Király *et al*. 2013; Lõhmus *et al*. 2023). Microclimate and competitors can be determining factors in the distribution of autotrophs in fine‐scale microhabitats (Ellis & Coppins 2006; Armstrong & Welch 2007; Hurtado *et al*. 2019).

Epiphytic organisms are specialized to use plants as substrates for growth (i.e., phorophytes) (Zotz 2016). Their vertical distribution is determined by microclimate gradients, such as light, temperature, and humidity (Zotz *et al*. 2001; Ellis 2012; Zotz 2016). However, niche occupation of epiphytes also occurs in the radial dimension, including mountain slopes, tree trunks, walls, rocks, and even ant‐hills (King 2003; Frasco & Dobson 2010; Taylor & Burns 2016). For instance, the radial distribution of some mistletoes (Loranthaceae), ferns (Aspleniaceae, Hymenophyllaceae, and Polypodiaceae), and orchids (Orchidaceae) on tree trunks is directional (Tremblay & Castro 2009; García‐González *et al*. 2016; Taylor & Burns 2016). This indicates a direction determined by subtle microclimate variations, even found around tree trunks. Such variations are driven by light intensity, where higher UV radiation, increased temperature, and reduced humidity restrict establishment and damage photosystems (Kranner *et al*. 2008; Ranius *et al*. 2008; Király *et al*. 2013; Taylor & Burns 2016). This is because light strikes the equator‐facing trunk directly because light reaches Earth more perpendicularly at low latitudes (Leege 2001; Golub & Pasachoff 2017). This creates restrictive microclimate conditions in equator‐facing parts of tree trunks compared to the opposite direction, that is, facing the poles, which are more shaded throughout the day, creating an optimal occurrence zone for some epiphytes (Tremblay & Castro 2009; García‐González *et al*. 2016; Taylor & Burns 2016; Lauriault & Wiersma 2020).

As the tree trunk microhabitat is continuous, the distribution of organisms contains directional data (i.e., compass‐derived) (Jammalamadaka & SenGupta 2001; Pewsey *et al*. 2013). This requires use of circular statistics that allow testing of hypotheses of directionality, comparing different circular samples/organisms, and describing important data, such as mean direction and aggregation. However, as far as we know, only nine plant species have been studied using this approach, including vascular epiphytes and mistletoes (Tremblay & Castro 2009; García‐González *et al*. 2016; Taylor & Burns 2016). This limits our understanding of general patterns and how other important groups of autotrophs are distributed on tree trunks. No assessment has yet been conducted for avascular epiphytes, such as lichens. These are symbioses of a fungus (mycobiont), usually dominant, and one or more algae or cyanobacteria (photobiont) (Nash 1996; Scharnagl *et al*. 2023). While the fungus provides structure, protection, water, and micronutrients to the photobiont, the photobiont provides photosynthates to the fungus, characterizing a service‐resource obligate mutualism (*sensu* Boucher *et al*. 1982). This symbiotic association forms a thallus, which is a self‐sufficient, and self‐propagating structure and among the most complex structures of the fungal kingdom (Nash 1996; Scharnagl *et al*. 2023). Lichen thalli have three main growth forms crustose, foliose, and fruticose (Nash 1996; Ellis 2012; Scharnagl *et al*. 2023). Most lichens belong to the order Ascomycota, being associated with Chlorophyta algae (Nash 1996).

Lichens are an interesting study group because they occur in more diverse and extreme habitats than vascular epiphytes (Zotz & Bader 2009; Després *et al*. 2012), being tolerant to extreme desiccation and exposure to UV light (Grime 1977; Nash 1996; Kranner *et al*. 2008). Lichesn are poikilohydric organisms (Nash 1996), although tolerant to dehydration, long periods in the desiccated state, makes them highly dependent on microclimatic conditions, especially humidity (Nash 1996; Kranner *et al*. 2008; Király *et al*. 2013; Matos *et al*. 2015). In the radial plane of the trunk microhabitat, lichens are expected to orient away from sunlight, that is, facing north in the northern hemisphere and south in the southern hemisphere (Ranius *et al*. 2008). Although this has been observed in lichens and mosses (Kraemer 1901; Gauslaa & Solhaug 2001; Ranius *et al*. 2008; Lauriault & Wiersma 2020; Esseen & Ekström 2023), accurate predictions using circular techniques would provide confirmation. In addition, lichen species often co‐occur in the same trunk microhabitat, with heterospecifics competiting for space (Lawrey 1991; Ranius *et al*. 2008; Ellis 2012; Matos *et al*. 2015). Thus, radial co‐occurrence patterns of distinct lichen species should be obtained by applying appropriate circular procedures. Finally, since factors, such as substrate height relative to the ground, phorophyte radial size, and canopy cover important in determining the occurrence of lichens (Ellis 2012; Király *et al*. 2013; Llewellyn *et al*. 2020), it is necessary to investigate if these important variables affect radial orientation. To address these, we studied crustose and foliose lichen distribution patterns around tree trunks in a habitat in the southern hemisphere, employing circular analytical methods.

We carefully chose a system with lichens inhabiting one species of palm tree because the stipe‐type trunk is close to circular, and using a single phorophyte allows standardization and avoids phylogenetic confounding effects related to different substrate surfaces. Since north‐facing sides of tree trunks in the southern hemisphere receive direct sunlight, creating harsh microclimate conditions, we hypothesize that (1) lichens will orient preferentially southward, where conditions are less stressful, that is, lower exposure to excess radiation, high temperatures, and low humidity. In addition, as both crustose and foliose lichens are susceptible to such restrictive conditions, we hypothesize that (2) the two lichen types will not differ in their preferred orientation, indicating that abiotic factors are more important than competitive interactions for space. As the restrictive cardinal microclimate conditions on trunks are determined by macrogeographic latitudinal patterns, leading to similar patterns in all trees at the same geographic location, we hypothesize that (3) other variables, such as lichen height relative to the ground, phorophyte radial size, and canopy cover, will not influence lichen radial orientation, which is governed by the azimuth (first hypothesis).

## MATERIAL AND METHODS

### Study site

**Fig. 1:**
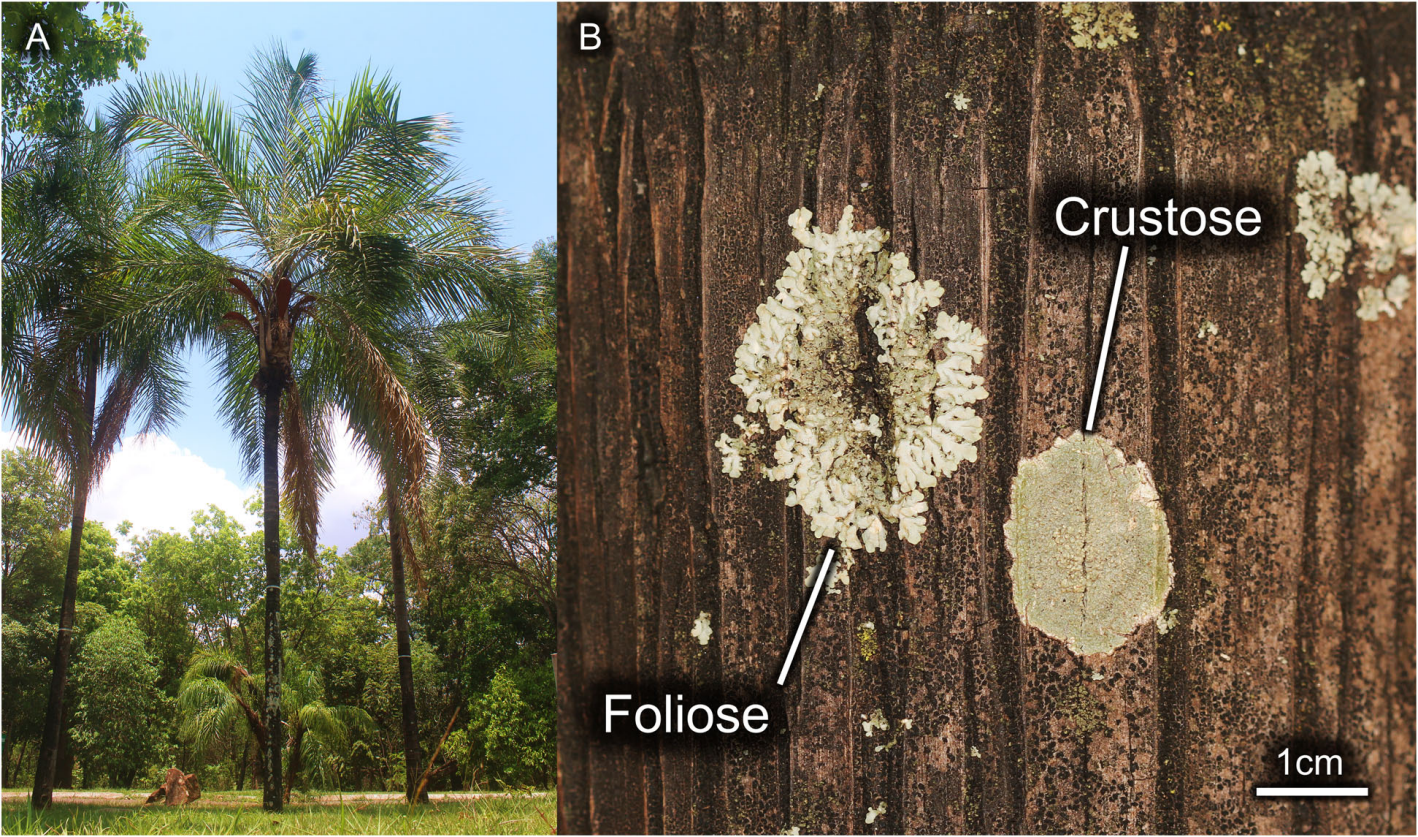
(A) Study site showing *Syagrus oleraceae* individuals; (B) detail of the two lichens, including *Lecanora* sp. (crustose type) and *Pyxine* sp. (foliose type).

The study was carried out in Caldas Novas State Park (PESCAN), in Goiás state, Brazil, in November 2022. The climate of the region is AW according to the Köppen‐Geiger classification, with hot, rainy summers (October to March) and warm, dry winters (April to September), with average annual rainfall of 1576 mm and average monthly temperature of 22.1°C (Alvares *et al*. 2013). The experiments were conducted at the entrance of the park, in an area of ca. 12,000 m^2^, composed of a few buildings immersed in a heterogeneous matrix of trees and lawns (17°46′07′′ S, 48°39′29′′ W; Fig. 1A). The main trees, from ca. 6 and 15 m in height, were the native *Copaifera langsdorffii*, *Dipteryx alata*, *Hymenaea courbaril* (Fabaceae), *Apeiba tibourbou* (Malvaceae), *Caryocar brasiliense* (Caryocaraceae), *Curatella americana* (Dilleniaceae), *Sterculia apetala* (Malvaceae), *Tabebuia aurea* (Bignoniaceae), and the exotic *Mangifera indica* (Anacardiaceae).

### Procedures

Substrate characteristics can affect microclimates of tree trunks and influence epiphyte occurrence, for example, roughness, porosity, and hardness (Kubiak & Osyczka 2020). To reduce interference from phylogenetic confounding effects related to different phorophyte species (see Ranius *et al*. 2008), we selected individuals of a single species: the native palm *Syagrus oleracea* (Mart.) Becc. (Arecaceae) (Fig. 1A). This is a perennial, single‐stemmed, large plant with evergreen and spiral leaves, which can reach heights of 10–20 m (Lorenzi *et al*. 2004). The stipe‐type trunk is erect, smooth, and concentric, with a regular circumference along its entire length, being close to a perfect cylinder, thus reducing azimuth biases.

We selected 20 *S. oleracea* individuals in the area, with heterogeneous distribution, including individuals close to each other (ca. 2 m), or >10 m from their nearest neighbour. Considering the overall area, tree density was low, as 0.002 individuals m^−2^. For each palm, we measured circumference at the breast height (1.50 m from the ground) then converted this into diameter at breast height (DBH). DBHs mean ± SD was 14.61 cm ± 1.95, ranging between 12.58 and 20.06 cm. We also estimated percentage canopy cover for each tree based on the mean extracted from the four main cardinal points (i.e., north, south, east, and west). We took photos of the canopy at 1 m height for each cardinal point then estimated percentage of canopy cover using the Canopeo^®^ software. Cardinal points were assessed using Compass^®^ software. The mean canopy cover around *S. oleracea* palms was 16.26% (±11.07%), between 2.61% and 42.27%.

We surveyed all lichen thalli present on tree trunks from the base to a height of 2 m from the ground. We chose this height threshold because it enables accurate measurements and contains most of the lichens, which were less common at higher points (Fig. 1A). Lichen thalli can be difficult to separate and visualize in the substrate because they can agglomerate and are very small. Thus, each lichen thallus, characterized by an isolated, circular, concentric structure with a diameter ≥2.0 cm, was considered a different individual. Lichen thalli were classified according to growth form: crustose or foliose. There were n fruticose lichens on the palm trunks. The azimuth of each lichen individual was recorded to an angulation accuracy of 1° (0° = North) using the Compass^®^ software. We also recorded height from the ground of each lichen individual. Since thalli were primarily circular and occasionally ellipsoid, azimuth and height measurements were taken using thalli centroids as reference for standardization.

### Statistical analyses

We used circular statistics because of the directional nature of lichen distribution around the tree trunks (Jammalamadaka & SenGupta 2001; Pewsey *et al*. 2013). Considering overall lichen distribution, we calculated descriptive circular statistics, including circular mean (*μ*_circ_), circular standard deviation (sd_circ_), and length of the mean vector (*r*). The latter represents the proportion of data clustering around *μ*_circ_, ranging from 0 (perfectly distributed around a circle) to 1 (perfectly concentrated) (Jammalamadaka & SenGupta 2001; Pewsey *et al*. 2013). We checked if the data followed von Mises distribution (i.e., the circular analog to normal distribution) using a Watson's test. As this was not Von Mises distributed, we applied the non‐parametric Hermans‐Rasson test to investigate if distribution departs from uniformity (Landler *et al*. 2019). The alternative hypothesis indicates that lichens are not distributed uniformly around the trunk, indicating occurrence peaks (Jammalamadaka & SenGupta 2001; Pewsey *et al*. 2013). Since our data are aggregated into bins due to recording to the nearest degree (n = 360), we used a variant of the Hermans‐Rasson test (i.e., a tie‐breaking version) that controls for the type I error associated with grouped data (adjusting 10,000 iterations) (Landler *et al*. 2020). Next, we conducted V‐tests to assess if the distribution coincides with that of a specified *μ*_circ_. We ran two complementary V‐tests: one testing if distribution coincides with south (i.e., 180°) and the other, if it coincides with north (i.e., 0°). According to our initial hypothesis, we expected non‐significant and significant effects, respectively. We also investigated lichen distribution at the level of palm trees using a Hermans‐Rasson test for each tree. Then, we used the trees in which lichens had a directional orientation (i.e., non‐uniform) to conduct a parametric Rayleigh test using *μ*_circs_ of these individual palms. We used this test because the data did not differ from the Von Mises distribution.

This same routine, including descriptive parameters, normality, uniformity, and specified direction tests, was conducted separately for each lichen type. For uniformity, we employed a Hermans‐Rasson test for the foliose type and a Rayleigh test for the crustose type. We did not conduct palm‐level analyses for the lichen types due to some small sample sizes and to avoid type I errors.

To address our second hypothesis, we investigated differences between crustose and foliose lichens. We first estimated overlap between distributions of the two lichen types by applying a circular Kernel density function, then calculating the overlap coefficient between the two functions, which ranges from 0 (complete non‐overlap) to 1 (complete overlap) (Oliveira‐Santos *et al*. 2013). Then we tested if the two lichen types differ in (1) circular distributions, (2) circular mean directions, or (3) circular concentrations (Landler *et al*. 2021). To test the first, we used MANOVA based on sines and cosines of the angles. Differences in mean directions were tested using the Rao polar test. Differences in concentrations of the two distributions were investigated using the Wallraff test of angular dispersion around *μ*_circ_.

To address our third hypothesis, we investigated whether, overall, the lichens follow the same direction throughout the whole range of heights investigated by fitting a linear‐circular regression (i.e., with a linear predictor and circular response variables) using the local‐linear estimator and 10,000 bootstrap resamples (Alonso‐Pena *et al*. 2021). This same procedure was repeated for each lichen type separately (i.e., crustose and foliose). We additionally investigated if the direction of lichens on palms varied according to canopy cover and palm DBH (linear predictors). We used the *μ*_circs_ of palms with lichen directional orientation as circular response variable and fitted linear‐circular regressions, adopting the same procedure described for height analyses. With palms as replicates, we also investigated the influence of canopy cover and DBH predictors on level of lichen aggregation using the *r* values as the response variable. Since these values are confined between 0 and 1, we fitted a GLM (Generalized Linear Model) adjusting the beta distribution with *logit* link, which is appropriate for proportional data (i.e., confined into bounds) (Douma & Weedon 2019).

Descriptive statistics, Watson tests, Rayleigh tests, V‐tests, and the Wallraff test were conducted using the R‐package *circular* v. 0.4.95 (Agostinelli & Lund 2022). Linear‐circular regressions were conducted in the R‐package *NPCirc* v. 3.1.1 (Oliveira *et al*. 2014). Smoothing parameters for plotting regression lines were calculated using the function *bw.reg.lin.circ*. Hermans‐Rasson tests were conducted using the R‐package *CircMLE* v. 0.3.0 (Fitak & Johnsen 2017) and the routine provided by Landler *et al*. (2020). Rao polar test was conducted using the R‐package *CircStats* v. 0.2.6 (Jammalamadaka & SenGupta 2001). The MANOVA was done using the R‐stats package. The GLM was built in the R‐package *glmmTMB* v. 1.1.4 (Brooks *et al*. 2017); model fit was checked by simulating residuals 1,000 times using R‐package *DHARMa* v. 0.4.6 (Hartig 2022); and model significance was assessed using type II Wald Chi‐square test in the R‐package *car* v. 3.1.2 (Fox & Weisberg 2019). All analyses were performed in R software v. 4.3.1 (R Core Team 2023). Data and codes from this study are openly available at: https://doi.org/10.6084/m9.figshare.28590416.v1.

## RESULTS

**Fig. 2:**
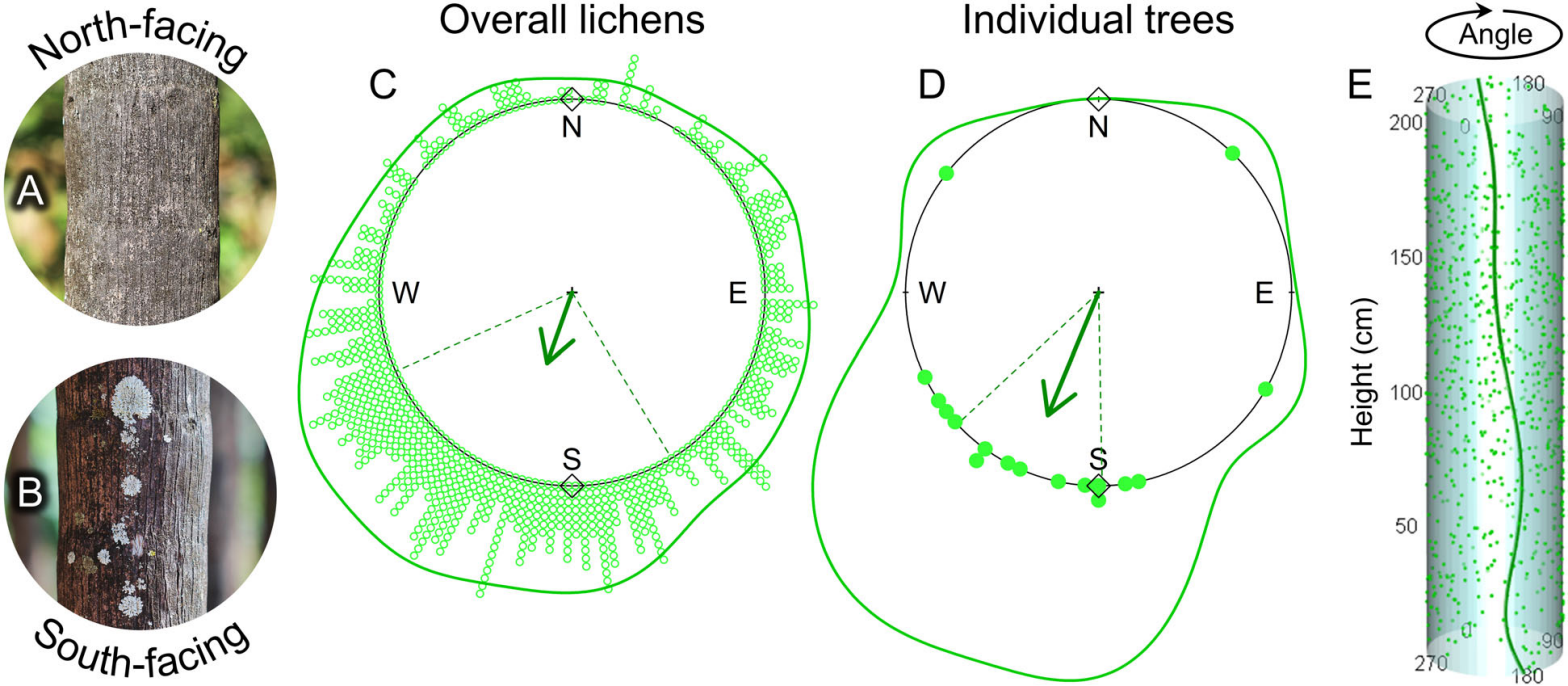
Lichen distributions on palm tree trunks. (A, B) North‐ and south‐facing trunks of the same tree demonstrate the occurrence of lichens and the different patterns of sunlight intensity. (C) Circular plot showing the distribution of overall lichens sampled, where each point refers to an individual lichen. (D) Circular plot showing analysis at the level of palm trees, where each point refers to the *μ*_circ_ of lichens distributed within a palm tree. In C and D, arrows point to *μ*_circ_ of the respective distribution, and thelength is proportional to the length of vector *r*. Dashed lines represent 25% and 75% quantiles. N, E, S, and W represent north (0°), east (90°), south (180°), and west (270°), respectively. Lines around plots represent Kernel density circular estimates of the distributions. Rhombuses detail north and south, the specified means by which the distributions were tested to be coincident. (E) Cylindrical representation of a tree trunk showing consistent lichen orientation along the height continuum. The line indicates predicted probability (non‐significant). Points in C–E show observed data.

We found 845 lichens on 19 out of the 20 palm trees. Of these, 665 were foliose (*Pyxine* sp., Ascomycota: Physciaceae), and 180 were crustose (*Lecanora* sp., Ascomycota: Lecanoraceae). Overall, lichens were not uniformly distributed around the trunks, indicating a preferred direction (*T* = 187.25, *P* < 0.001; Fig. 2A–C). Lichens were oriented towards *μ*_circ_ of 199.56° (±78.91 sd_circ_), with a moderate aggregation of 0.39 (i.e., length of the vector *r*). Considering the hypothesis of orientation towards specific directions, the lichen distribution coincided significantly with *μ*_circ_ 180° (south) and was not coincident with 0° (north) (*Z* = 112.58, *P* < 0.001; *Z* = 112.58, *P* = 1.00, respectively; Fig. 2A–C). Analysis at the level of palm trees produced similar patterns. Seventeen of 19 trees had lichens non‐uniformly distributed (89.5%; all *P*s ≤ 0.05). The two trees without lichen directionality had only 11 and 22 lichen thalli (*P* = 0.26 and *P* = 0.09, respectively), which was too small a sample size to indicate significant directionality. At the level of palm trees, *μ*_circs_ were also not uniformly distributed (*Z* = 8.04, *P* < 0.001; Fig. 2D). The orientation was towards a *μ*_circ_ of 202.54 (±49.57), with higher aggregation of 0.69. Fo specific orientation, we found that the mean distribution of lichens at tree level also coincided significantly with *μ*_circ_ 180° (south) and was not coincident with 0° (north) (*Z* = 6.86, *P* < 0.001; and *Z* = 6.86, *P* = 1.00; Fig. 2D).

**Fig. 3:**
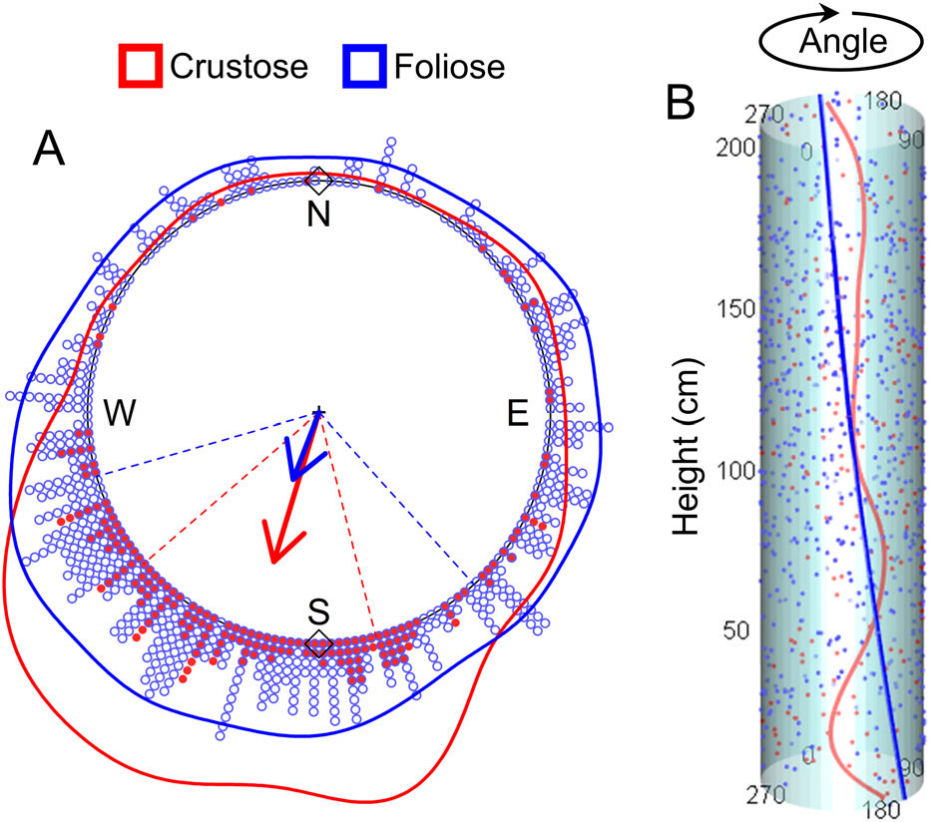
(A) Circular plot showing distribution of the two lichen types. Arrows point to the *μ*_circs_ of the respective distributions, and their length is proportional to the length of the vector *r*. Dashed lines represent 25% and 75% quantiles. N, E, S, and W represent north (0°), east (90°), south (180°), and west (270°), respectively. Lines around the plot represent Kernel density circular estimates of the distributions. Rhombuses detail north and south, the specified means by which the distributions were tested to be coincident. (B) Cylindrical representation of a tree trunk shows consistent lichen orientation of both types along the height continuum. The line indicates predicted probabilities (non‐significant). Points in A and B show observed data.

Considering lichen types, distributions of both crustose and foliose lichens were not uniform around tree trunks (*Z* = 384.32, *P* < 0.001; and *T* = 95.10, *P* < 0.001; Fig. 3A). When testing the orientation towards specific means, both crustose and foliose lichens coincided significantly with *μ*_circ_ 180° (south) (*Z* = 352.09, *P* < 0.001; and *Z* = 70.62, *P* < 0.001, respectively; Fig. 3A); their directions were not significantly coincident with 0° (north) (*Z* = 352.09, *P* = 1.00; and *Z* = 70.62, *P* = 1.00, respectively; Fig. 3A).

The two lichen distributions had an overlap of 0.68, indicating a dissimilarity of 0.32. In agreement, we did not find differences between crustose and foliose lichens in terms of their overall distributions (*F*_1,843_ = 0.68, *P* = 0.51; Fig. 3A). The crustose type had *μ*_circ_ 196.81° (±50.86), which was similar and not significantly different from *μ*_circ_ 201.18° (± 87.68) of the foliose type (*H* = 0.11, *P* = 0.74; Fig. 3A). However, there were significant differences in concentration (*χ* ^2^ = 45.52, *P* < 0.001; Fig. 3A), with crustose being more aggregated than foliose lichens, the latter having a more widespread distribution (*r* = 0.67 vs. 0.31, respectively).

The potential influence of height was ruled out in all regression analyses. There was no significant effect of height on overall lichen direction (*C* = 0.03; *P* = 0.16; Fig. 2E). This indicates that the orientation is consistent and does not change along the trunk, at least up to the analysed height of 2 m (i.e., lichens do not rotate along the trunk). The same lack of influence of height on direction was observed for the lichen types, both crustose and foliose (*C* = 0.24; *P* = 0.08; and *C* = 0.02; *P* = 0.32, respectively; Fig. 3B). The linear‐circular regression lines corroborate this consistent orientation along the height continuum (Figs 2E and 3B).

Canopy cover and DBH did not significantly affect lichen direction across palm trees (*C* = 0.08, *P* = 0.51; *C* = 0.33, *P* = 0.44). These predictor variables also did not significantly influence the aggregation of lichens around trunks (i.e., *r* values) (canopy cover: *χ* ^2^ = 0.19, *P* = 0.66; DBH: *χ* ^2^ = 0.49, *P* = 0.49).

## DISCUSSION

### Overview

Several facets of lichen occupation at the radial dimension of the trunk microhabitat were explored. Choosing a palm tree as model of a trunk close to a cylinder allowed us to test hypotheses while reducing biases of substrate irregularities and the phylogenetic influence of different phorophytes. Lichens of our study system were not uniformly distributed at the fine scale of trunk; they oriented preferentially southward, where there is less incident sunlight and a more favourable microclimate. This pattern is consistent across palm trees and for both lichen types, foliose and crustose. Although having the same directionality, crustose lichens had a more aggregated distribution, indicating different tolerances to extremes away from the optimal radial growth zone. South orientation is also consistent across variations in lichen height above the ground and local characteristics, including canopy cover and radial trunk size. As far as we know, this is the first time such patterns have been reported for lichens. However, caution must be taken when extrapolating these results as they refer to a population of one phorophyte and involve only two lichen species of two growth forms.

### Hypothesis 1

Our results demonstrated that the studied lichens are not uniformly distributed around the trunks, with a preferential southward orientation. This indicates an optimal radial occurrence zone on trunks on the south‐facing side in our study site in the southern hemisphere, corroborating our first hypothesis. Tese findings agree with observations in the northern hemisphere, where lichens and mosses commonly orient preferentially northward (Gauslaa & Solhaug 2001; Ranius *et al*. 2008; Frasco & Dobson 2010; Lauriault & Wiersma 2020). Since there is more direct sunlight incidence in the north‐facing portion of trees in the southern hemisphere (Golub & Pasachoff 2017), microclimate conditions also change accordingly, including stressors such as increased UV intensity, higher temperature, and lower humidity in this portion (Király *et al*. 2013). Thus, even though lichens have adaptations that allow them to cope with such stressors (Grime 1977; Nash 1996; Kranner *et al*. 2008), the northernmost trunk portions have higher stress levels while the southernmost trunk provides better establishment conditions. These results show how distribution of lichens varies at microhabitat level at the fine scale of the radial dimension of trunks.

Lichen propagules are light, long‐range structures that can be dispersed through rainwater (Nash 1996; Després *et al*. 2012). In our system, this dispersal mode tends to perpetuate the patterns since vertical water runoff will transport propagules to lower trunk parts, probably without major changes in azimuth. However, the main dispersal mode of lichen propagules is via wind (Nash 1996; Després *et al*. 2012; Llewellyn *et al*. 2020), which blow in all directions in our studied environment (Weather Spark 2022), indicating that propagules can potentially reach all portions of the tree trunk. This is corroborated by the occurrence of lichens throughout the studied trunks despite the preferred direction. The dispersal of sessile organisms involves randomness, where reaching appropriate and inappropriate locations establishment success (Fagerström 1988). Thus, the observed south orientation reflects the greater establishment success within an optimal radial occurrence zone. Overall, lichen distributions showed 39% aggregation, indicating that despite distribution having a specific direction, they were still distributed beyond the specific average direction. This suggests they can tolerate other parts of the circumference but that their distribution is severely limited in the north‐facing trunk that receives the most direct sunlight. It is likely that as some of studied lichens are closer to facing north, they become progressively more stressed. For example, excess light damages lichen photosystems (Zotz & Bader 2009). However, aggregation increased to 69% when the average tree direction was considered, confirming that preferred directions tend to converge southward across all trees surveyed.

As the investigated system only assessed a few species in restricted spatial conditions, although the patterns found were consistent, lichen diversity is high and may involve other patterns as found in other habitats and conditions, for example, different substrates, latitudes, humidity and light regimes. Some lichen species grow preferentially in direct sunlight (Nash 1996; Ellis 2012); thus, our results should be extrapolated with caution.

### Hypothesis 2

We also confirmed our second hypothesis, that the studied lichen types do not differ in their preferred orientation. Crustose and foliose lichens had similar distributions and oriented preferentially towards the south and away from the north, with mean directions being similar (*μ*_circs_ 196.81° and 201.18°, respectively). These patterns confirm the consistent cardinal effects of microclimate conditions around trunks. They also suggest that the impact of the microenvironmental filter is more determinant on lichen distribution than that of the interaction filter.

Although interspecific competition occurs in lichen communities, different lichen species can occupy the same microenvironment, including tree trunks (Armstrong & Welch 2007; Ellis 2012). The restrictive microclimatic conditions (high light incidence in the north) probably induce a similar response in both lichens aggregate and overlap their distributions in the same spatial/radial niche. This coexistence suggests they probably differ in other niche axes because niche partitioning in lichens can be very subtle (Ellis 2012). We found that distribution of the crustose lichen was more aggregated than that of the foliose type. This may indicate that the crustose lichen is more sensitive to extremes beyond its optimum radial zone, suggesting higher tolerance of the foliose lichen, with a wider radial niche breadth, potentially reducing competition for space between the two species. Crustose lichens are more common in the most humid areas, while foliose lichens can resist desiccation and are associated with more arid areas (Matos *et al*. 2015). In addition, crustose lichens are less competitive, often over‐grown by foliose lichens and bryophytes, generally following a succession process (Ellis & Coppins 2006; Armstrong & Welch 2007; Ranius *et al*. 2008). This less tolerance to dryness and reduced competitive ability probably explains the narrower radial occurrence and lower abundance of crustose lichens. However, it is important to recognize that our results refer to only two growth forms. Thus, we do not know whether non‐segregative orientation occurs among other co‐occurring species, especially whether pole‐facing orientation also applies to fruticose lichens.

### Hypothesis 3

The microhabitat heterogeneity, including canopy cover and phorophyte size, influences the distribution of lichens because it suggests different requirements (Ellis 2012; Esseen & Ekström 2023). However, the height of the lichens up to 2 m from ground level did not influence their radial orientation, which remained consistent throughout the whole vertical extension of the trunks for lichens of both types. At palm tree level, there was no effect of canopy cover or phorophyte radial size (DBH) either on mean direction or aggregation of lichens. The lack of influence of these factors on lichen orientation corroborates our third hypothesis, indicating that, in a given geographic region, the relative cardinal position on the trunk governs the radial distribution of the studied lichens, without effects of other variables. These results are confined to a height limit of 2 m, which contained most of the studied lichens. Thus, the effect of these predictor variables may be important on the radial distribution of other lichens, especially at greater heights, as commonly occurs in tropical forests.

### Limitations, implications and future “directions”

Lichens are distributed worldwide and occur in all terrestrial ecosystems. Thus, based on the marked effect of azimuth, other geographic locations could have different idiosyncratic differences, depending on habitat and ecological conditions, which will probably involve different light incidence and will influence both direction and aggregation patterns. For example, at latitude 0, all parts of a trunk can receive light equally, while at very high latitudes, the part facing the equator may be most suitable due to lower light availability when approaching the poles. Furthermore, orientation patterns may depend on epiphyte species, which may be adapted to sunny environments, have partitioned the niche with other species, or even have been competitively excluded from a given part of the radial plane, as found on rocks (Armstrong & Welch 2007; García‐González *et al*. 2016; Taylor & Burns 2016). Future studies should test the same predictions as investigated here to confirm the generality of our findings. These may include (1) the influence of azimuth on lichen size, (2) other lichen species and growth forms such as the fruticose type, (3) other locations at different latitudes and biomes, and (4) other phorophytes with distinct characteristics. In addition, (5) physiological studies comparing lichens facing different cardinal points could provide insights into the stress level posed by radial orientation using, for instance, chlorophyll and photosynthesis measurements. Finally, experimental studies could include (6) testing the success of propagule colonization at different cardinal points, and (7) rotating colonized structures such as trunk pieces to other cardinal directions and monitoring the growth and survival of lichens.

Furthermore, our study constitutes an important bridge between epiphyte ecology and circular analytical methods. As far as we know, this is the first investigation of lichen radial distribution using these procedures. In addition, we demonstrate that this can be a valuable tool for testing biological hypotheses using valuable tests that are less known to biologists, involving specific preferred direction assessments, between‐sample comparisons, and linear‐circular relations. Our findings provide insights into lichen biology and how abiotic factors modulate their distribution on a fine scale.

## CONCLUSION

We demonstrate that lichens preferentially orient southward in the southern hemisphere, which constitutes an optimal radial occurrence zone located in the opposite direction to direct sunlight incidence, creating more stressful microclimatic conditions such as more UV light, increased temperatures, and reduced humidity. The southward orientation pattern was consistent for crustose and foliose lichens that also had similar distributions, indicating that the effects of radial distribution of microclimate conditions are a larger determinant than potential competition for the radial spatial niche. Finally, azimuth was the main factor governing the radial distribution of the studied lichens, which were unaffected by height from the ground, canopy cover, or phorophyte size. In summary, our study shows how abiotic conditions are determinants of the niche occupation of autotrophs, even around tree trunks. This opens several possibilities for hypothesis testing that will benefit our understanding of how planetary‐scale factors indirectly influence organism distribution at subtle microscales.

## AUTHOR CONTRIBUTIONS

JCFC conceived and designed the study. All authors collected the data. JCFC analysed the data. BBSN and JCFC wrote the first draft of the manuscript. All authors approved the final version of the paper.

## References

[plb70055-bib-0001] Agostinelli C. , Lund U. (2022) R package ‘circular’: circular statistics. R‐package v. 0.4‐95. https://CRAN.R‐project.org/package=circular

[plb70055-bib-0002] Alonso‐Pena M. , Ameijeiras‐Alonso J. , Crujeiras R.M. (2021) Nonparametric tests for circular regression. Journal of Statistical Computation and Simulation, 91, 477–500.

[plb70055-bib-0003] Alvares C.A. , Stape J.L. , Sentelhas P.C. , Gonçalves J.D.M. , Sparovek G. (2013) Köppen's climate classification map for Brazil. Meteorologische Zeitschrift, 22, 711–728.

[plb70055-bib-0004] Armstrong R.A. , Welch A.R. (2007) Competition in lichen communities. Symbiosis, 43, 1–12.

[plb70055-bib-0005] Ashraf M.H.P.J.C. , Harris P.J. (2013) Photosynthesis under stressful environments: an overview. Photosynthetica, 51, 163–190.

[plb70055-bib-0006] Boucher D.H. , James S. , Keeler K.H. (1982) The ecology of mutualism. Annual Review of Ecology and Systematics, 13, 315–347.

[plb70055-bib-0007] Brooks M.E. , Kristensen K. , Van Benthem K.J. , Magnusson A. , Berg C.W. , Nielsen A. , Skaug H.J. , Machler M. , Bolker B.M. (2017) glmmTMB balances speed and flexibility among packages for zero‐inflated generalized linear mixed modeling. The R Journal, 9, 378–400.

[plb70055-bib-0008] Després V. , Huffman J.A. , Burrows S.M. , Hoose C. , Safatov A. , Buryak G. , Fröhlich‐Nowoisky J. , Elbert W. , Andreae M. , Pöschl U. , Jaenicke R. (2012) Primary biological aerosol particles in the atmosphere: a review. Tellus B: Chemical and Physical Meteorology, 64, 15598.

[plb70055-bib-0009] Douma J.C. , Weedon J.T. (2019) Analysing continuous proportions in ecology and evolution: a practical introduction to beta and Dirichlet regression. Methods in Ecology and Evolution, 10, 1412–1430.

[plb70055-bib-0010] Ellis C.J. (2012) Lichen epiphyte diversity: a species, community and trait‐based review. Perspectives in Plant Ecology, Evolution and Systematics, 14, 131–152.

[plb70055-bib-0011] Ellis C.J. , Coppins B.J. (2006) Contrasting functional traits maintain lichen epiphyte diversity in response to climate and autogenic succession. Journal of Biogeography, 33, 1643–1656.

[plb70055-bib-0012] Esseen P.A. , Ekström M. (2023) Influence of canopy structure and light on the three‐dimensional distribution of the iconic lichen *Usnea longissima* . Forest Ecology and Management, 529, 120667.

[plb70055-bib-0013] Fagerström T. (1988) Lotteries in communities of sessile organisms. Trends in Ecology & Evolution, 3, 303–306. 21227263 10.1016/0169-5347(88)90106-1

[plb70055-bib-0014] Fitak R.R. , Johnsen S. (2017) Bringing the analysis of animal orientation data full circle: model‐based approaches with maximum likelihood. Journal of Experimental Biology, 220, 3878–3882. 28860118 10.1242/jeb.167056 PMC6514460

[plb70055-bib-0015] Fox J. , Weisberg S. (2019) An {R} companion to applied regression, 3rd edition. Sage, Thousand Oaks, USA, pp 608.

[plb70055-bib-0016] Frasco M. , Dobson C. (2010) Moss, beech trees, and stemflow: Integrated science. MSTA Journal, 55, 27–35.

[plb70055-bib-0017] García‐González A. , Damon A. , Riverón‐Giró F.B. , Ávila‐Díaz I. (2016) Circular distribution of three species of epiphytic orchids in shade coffee plantations, in Soconusco, Chiapas, Mexico. Plant Ecology and Evolution, 149, 189–198.

[plb70055-bib-0018] Gauslaa Y. , Solhaug K.A. (2001) Fungal melanins as a sun screen for symbiotic green algae in the lichen *Lobaria pulmonaria* . Oecologia, 126, 462–471. 28547230 10.1007/s004420000541

[plb70055-bib-0019] Golub L. , Pasachoff J.M. (2017) The sun. Reaktion Books, London, UK, pp 224.

[plb70055-bib-0020] Grime J.P. (1977) Evidence for the existence of three primary strategies in plants and its relevance to ecological and evolutionary theory. The American Naturalist, 111, 1169–1194.

[plb70055-bib-0021] Hartig F. (2022) Dharma: residual diagnostics for hierarchical (multi‐level/mixed) regression models. R‐package v. 0.4.6. https://CRAN.R‐project.org/package=DHARMa

[plb70055-bib-0022] Hurtado P. , Prieto M. , Aragón G. , Escudero A. , Martínez I. (2019) Critical predictors of functional, phylogenetic and taxonomic diversity are geographically structured in lichen epiphytic communities. Journal of Ecology, 107, 2303–2316.

[plb70055-bib-0023] Jammalamadaka S.R. , SenGupta A. (2001) Topics in circular statistics, Vol. 5. World Scientific, Washington, DC, pp 336.

[plb70055-bib-0024] King T.J. (2003) Mosses and aspect; why is *Scleropodium purum* abundant on the north‐facing sides of ant‐hills? Journal of Bryology, 25, 211–213.

[plb70055-bib-0025] Király I. , Nascimbene J. , Tinya F. , Ódor P. (2013) Factors influencing epiphytic bryophyte and lichen species richness at different spatial scales in managed temperate forests. Biodiversity and Conservation, 22, 209–223.

[plb70055-bib-0026] Kraemer H. (1901) The position of Pleurococcus and mosses on trees. Botanical Gazette, 32, 422–423.

[plb70055-bib-0027] Kraft N.J. , Adler P.B. , Godoy O. , James E.C. , Fuller S. , Levine J.M. (2015) Community assembly, coexistence and the environmental filtering metaphor. Functional Ecology, 29, 592–599.

[plb70055-bib-0028] Kranner I. , Beckett R. , Hochman A. , Nash T.H. (2008) Desiccation‐tolerance in lichens: a review. The Bryologist, 111, 576–593.

[plb70055-bib-0029] Kubiak D. , Osyczka P. (2020) Non‐forested vs forest environments: the effect of habitat conditions on host tree parameters and the occurrence of associated epiphytic lichens. Fungal Ecology, 47, 100957.

[plb70055-bib-0030] Landler L. , Ruxton G.D. , Malkemper E.P. (2019) The Hermans‐Rasson test as a powerful alternative to the Rayleigh test for circular statistics in biology. BMC Ecology, 19, 1–8. 31391040 10.1186/s12898-019-0246-8 PMC6686250

[plb70055-bib-0031] Landler L. , Ruxton G.D. , Malkemper E.P. (2020) Grouped circular data in biology: advice for effectively implementing statistical procedures. Behavioral Ecology and Sociobiology, 74, 1–8. 10.1007/s00265-020-02881-6 PMC7373216 32728310

[plb70055-bib-0032] Landler L. , Ruxton G.D. , Malkemper E.P. (2021) Advice on comparing two independent samples of circular data in biology. Scientific Reports, 11, 20337. 34645855 10.1038/s41598-021-99299-5 PMC8514454

[plb70055-bib-0033] Lauriault P. , Wiersma Y.F. (2020) Identifying important characteristics for critical habitat of boreal felt lichen (*Erioderma pedicellatum*) in Newfoundland, Canada. The Bryologist, 123, 412–420.

[plb70055-bib-0034] Lawrey J.D. (1991) Biotic interactions in lichen community development: a review. The Lichenologist, 23, 205–214.

[plb70055-bib-0035] Leege L.M. (2001) Would you trust a bryophyte for directions? The American Biology Teacher, 63, 337–339.

[plb70055-bib-0036] Llewellyn T. , Gaya E. , Murrell D.J. (2020) Are urban communities in successional stasis? A case study on epiphytic lichen communities. Diversity, 12, 330.

[plb70055-bib-0037] Lõhmus A. , Motiejūnaitė J. , Lõhmus P. (2023) Regionally varying habitat relationships in lichens: the concept and evidence with an emphasis on north‐temperate ecosystems. Journal of Fungi, 9, 341. 36983509 10.3390/jof9030341 PMC10056719

[plb70055-bib-0038] Lorenzi H. , Souza H.M.D. , Costa J.T.M. , Cerqueira L.S.C.D. , Ferreira E.J.L. (2004) Palmeiras brasileiras e exóticas cultivadas. Plantarum Press, Nova Odessa, Brazil, pp 416.

[plb70055-bib-0039] Matos P. , Pinho P. , Aragon G. , Martínez I. , Nunes A. , Soares A.M. , Branquinho C. (2015) Lichen traits responding to aridity. Journal of Ecology, 103, 451–458.

[plb70055-bib-0040] Nash T.H. (1996) Lichen biology. Cambridge University Press, New York, NY, pp 502.

[plb70055-bib-0041] Oliveira M. , Crujeiras R.M. , Rodríguez‐Casal A. (2014) NPCirc: an R package for nonparametric circular methods. Journal of Statistical Software, 61, 1–26.

[plb70055-bib-0042] Oliveira‐Santos L.G.R. , Zucco C.A. , Agostinelli C. (2013) Using conditional circular kernel density functions to test hypotheses on animal circadian activity. Animal Behaviour, 85, 269–280.

[plb70055-bib-0043] Pewsey A. , Neuhäuser M. , Ruxton G.D. (2013) Circular statistics in R. Oxford University Press, Oxford, UK, pp 208.

[plb70055-bib-0044] R Core Team (2023) R: a language and environment for statistical computing. R Foundation for Statistical Computing, Vienna, Austria R v. 4.3.1. https://www.R‐project.org/

[plb70055-bib-0045] Ranius T. , Johansson P. , Berg N. , Niklasson M. (2008) The influence of tree age and microhabitat quality on the occurrence of crustose lichens associated with old oaks. Journal of Vegetation Science, 19, 653–662.

[plb70055-bib-0046] Scharnagl K. , Tagirdzhanova G. , Talbot N.J. (2023) The coming golden age for lichen biology. Current Biology, 33, R512–R518. 37279685 10.1016/j.cub.2023.03.054

[plb70055-bib-0047] Taylor A. , Burns K. (2016) Radial distributions of air plants: a comparison between epiphytes and mistletoes. Ecology, 97, 819–825. 27220198 10.1890/15-1322.1

[plb70055-bib-0048] Tremblay R.L. , Castro J.V. (2009) Circular distribution of an epiphytic herb on trees in a subtropical rain forest. Tropical Ecology, 50, 211.

[plb70055-bib-0049] Weather Spark (2022) The weather anywhere on Earth all year round. https://pt.weatherspark.com/

[plb70055-bib-0050] Zotz G. (2016) Plants on plants ‐ the biology of vascular epiphytes. Springer, Berlin, Germany, pp 297.

[plb70055-bib-0051] Zotz G. , Bader M.Y. (2009) Epiphytic plants in a changing world‐global: change effects on vascular and non‐vascular epiphytes. In: Lüttge U. , Cánovas F.M. , Pretzsch H. , Risueño M. , Leuschner C. (Eds), Progress in botany. Springer, Berlin, Germany, pp 147–170.

[plb70055-bib-0052] Zotz G. , Thomas V. , Hartung W. (2001) Ecophysiological consequences of differences in plant size: abscisic acid relationships in the epiphytic orchid *Dimerandra emarginata* . Oecologia, 129, 179–185. 28547595 10.1007/s004420100714

